# Adjuvant Chemotherapy for the Completely Resected Stage IB Nonsmall Cell Lung Cancer: A Systematic Review and Meta-Analysis

**DOI:** 10.1097/MD.0000000000000903

**Published:** 2015-06-05

**Authors:** Jiaxi He, Jianfei Shen, Chenglin Yang, Long Jiang, Wenhua Liang, Xiaoshun Shi, Xin Xu, Jianxing He

**Affiliations:** From the Department of Cardiothoracic Surgery (JH, JS, CY, LJ, WL, XS, XX, JH), the First Affiliated Hospital of Guangzhou Medical University; and Guangzhou Institute of Respiratory Disease & China State Key Laboratory of Respiratory Disease (JH, JS, CY, LJ, WL, XS, XX, JH), Guangzhou, China.

## Abstract

Adjuvant chemotherapy is recommended for postoperative stage II-IIIB nonsmall cell lung cancer patients. However, its effect remains controversial in stage IB patients. We, therefore, performed a meta-analysis to compare the efficacy of adjuvant chemotherapy versus surgery alone in stage IB patients.

Six electronic databases were searched for relevant articles. The primary and secondary outcomes were overall survival (OS) and disease-free survival (DFS). The time-to-event outcomes were compared by hazard ratio using log-rank test.

Sixteen eligible trials were identified. A total of 4656 patients were included and divided into 2 groups: 2338 in the chemotherapy group and 2318 in the control group (surgery only). Patients received platinum-based therapy, uracil-tegafur, or a combination of them. Our results demonstrated that patients can benefit from the adjuvant chemotherapy in terms of OS (HR 0.74 95% CI 0.63–0.88) and DFS (HR 0.64 95% CI 0.46–0.89). Patients who received 6-cycle platinum-based therapy (HR 0.45 95% CI 0.29–0.69), uracil-tegafur (HR 0.71 95% CI 0.56–0.90), or a combination of them (HR 0.51 95% CI 0.36–0.74) had better OS, but patients who received 4 or fewer cycles platinum-based therapy (HR 0.97 95% CI 0.85–1.11) did not. Moreover, 6-cycle platinum-based therapy (HR 0.29 95% CI 0.13–0.63) alone or in combination with uracil-tegafur (HR 0.44 95% CI 0.30–0.66) had advantages in DFS. However, 4 or fewer cycles of platinum-based therapy (HR 0.89 95% CI 0.76–1.04) or uracil-tegafur alone (HR 1.19 95% CI 0.79–1.80) were not beneficial.

Six-cycle platinum-based chemotherapy can improve OS and DFS in stage IB NSCLC patients. Uracil-tegafur alone or in combination with platinum-based therapy is beneficial to the patients in terms of OS, but uracil-tegafur seems to have no advantage in prolonging DFS, unless it is administered with platinum-based therapy.

## Abstract 2

Supplemental Digital Content is available in the text

## INTRODUCTION

Roughly 1.5 million new cases of lung cancer are diagnosed worldwide each year^1^ with nonsmall cell lung cancers (NSCLCs) accounting for about 85% of all reported cases. Though surgery is regarded as the primary treatment modality for early stage NSCLC, only 20% to 25% of the tumors are suitable for potentially curative resection, and a substantial percentage of these patients eventually develop local recurrence or distant metastases. As a result, more effective treatment strategies to reduce lung cancer mortality and recurrence rates are needed.

Five-year survival improvements of 5% to 10% have been reported with cisplatin-based adjuvant chemotherapy from multiple large randomized clinical trials^2–5^ and meta-analyses.^6,7^ Most of the randomized clinical trials reported positive results in patients with completely resected stage IB, II, and IIIA NSCLC.^2–5^ Only 1 large randomized trial CALGB9633^8^ focused on completely resected stage IB (T2N0) patients. However, its final results of overall survival (OS) and disease-free survival (DFS) lacked statistical significance.

Currently, the role of adjuvant cisplatin-based chemotherapy has been established by multiple large randomized phase III trials for resected stage II and IIIA NSCLC, but its role is controversial in stage IB patients. We, therefore, carried out a systematic review and meta-analysis to provide more reliable and up-to-date evidence on the effect of postoperative chemotherapy in stage IB patients through OS and DFS to identify whether the effect varies by patient subgroup. This included trying to verify the effects of different regimens and duration of postoperative chemotherapy.

## materials and methods

### Search Strategy

The electronic search was performed using PubMed, Medline, Cochrane Central Register of Controlled Trial, Cochrane Database of Systematic Reviews, ACP Journal Club, and Database of Abstracts of Reviews of Effects from the date of the earliest publication (1962) to October 2014. In order to achieve the maximum sensitivity, we used the following search strategy: “lung cancer” [all fields] AND (“chemotherapy, adjuvant” [MeSH Terms] OR “postoperative chemotherapy” [all fields]. All the articles were filtered by inclusion and exclusion criteria. The study did not involve any experiment on humans or animals, thus the ethical approval was not necessary.

### Inclusion and Exclusion Criteria

Only studies that investigated lung cancer patients who received radical resection with or without adjuvant chemotherapy were eligible for inclusion in our meta-analysis. Patients who received postoperative radiotherapy, preoperative chemoradiotherapy, or any other antitumor treatments were not included. The primary outcome was OS-defined as the time between the date of randomization and death or the last date of follow-up. The secondary outcome was DFS-defined as the time from randomization to the first date of recurrence or death. All publications were limited to human subjects and in English language. Case reports, expert opinions, abstracts, conference presentations, guidelines, and reviews were excluded in case of publication bias or data duplication. Publications with no primary or secondary outcomes, less than 2 treatment arms and the studies containing less than 20 patients in each treatment group were also excluded. When duplicated data were encountered, only the most novel and complete reports were included for data extraction and assessment.

### Data Extraction

All the data were independently extracted from the articles, tables, figures, and supplement of the publications by 3 inspectors (L.J., X.S., C.Y.). Discrepancies between reviewers were resolved by the discussion and consensus with the senior investigators (J.H., J.S.). The publication characteristics and time-to-event data including median overall survival hazard ratio (HR), disease-free survival HR, and 95% confidence interval (CI) were reviewed and extracted. Some of the included publications did not report the HR directly but they reported Kaplan–Meier curve or its *P* value of log-rank test between the treatment arms. As a result, we extracted the data of observed events number and the *P* value of HR. Some studies included stage I-III patients. According to the inclusion criteria, only the data of stage I patients were eligible for extraction. If the extraction of stage I data was unable to be finished or the raw data were not available, the study was excluded.

### Statistical Analysis

A systemic review and meta-analysis were performed to compare the postoperative chemotherapy to surgery alone by estimating the HR of OS and DFS. The data for analysis were categorical. Based on the null hypothesis stating that the frequency distribution of certain events observed in a sample were consistent with a particular theoretical distribution, χ^2^ tests were conducted to access the fit of distribution.^9,10^ In order to minimize this interference induced by the number of studies, I^2^ test was used to estimate the variation across the studies instead of the Q test,^10^ because Q test results were closely related to the number of included studies. The I^2^ was calculated by the formula: *I2* = 100% × (*Q*−*df*)*Q*; *Q* stood for a heterogeneity statistic and *df* was defined as the degree of freedom (*df* = total number of trials − 1). The heterogeneity was defined as low (25%–49%), moderate (50%–74%), or severe (>75%). Fixed-effect analysis model was used to calculate the HR. If the heterogeneity was severe, a random-effects analysis model would be used. In addition, the sensitivity test or subgroup analysis would be performed. Z-test was performed to calculate the *P* value, which was 2-sided and defined as statistically different when *P* < 0.05. The statistical analysis was conducted via Review Management 5.2 and Stata 12. The publication bias was analyzed via Stata 12. It would be considered insignificant when the *P* > 0.05 in both Egger and Begg tests.^11,12^

## RESULTS

**FIGURE 1:**
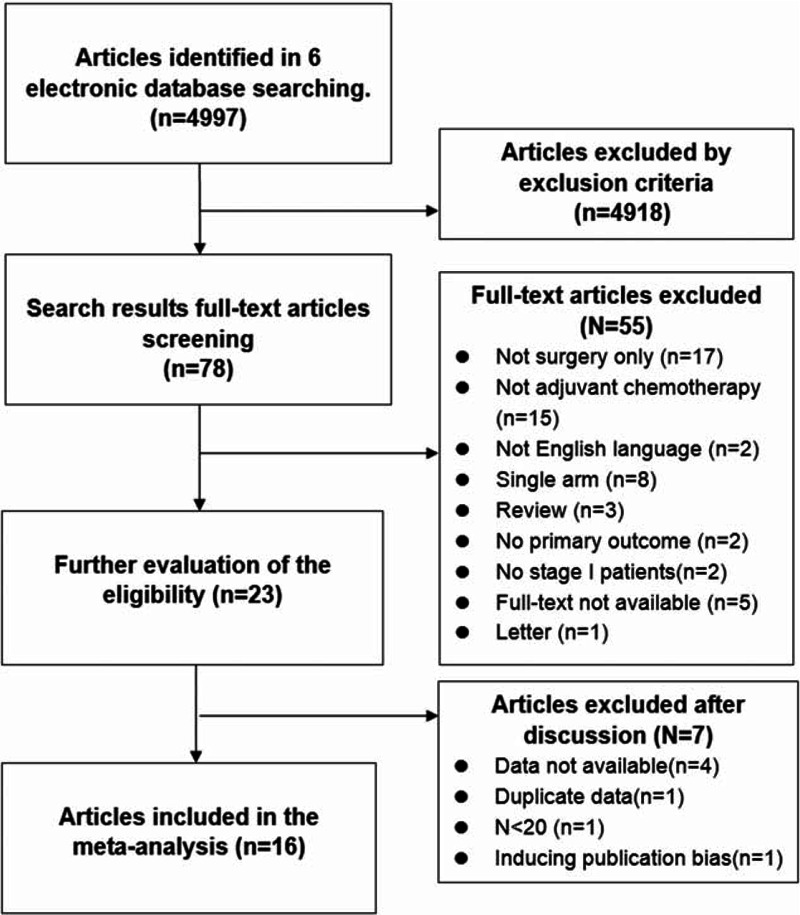
Selection and evaluation process of the eligible studies in the meta-analysis.

A search of 6 electronic databases revealed a total of 4997 potential articles for analysis; of these articles, 4918 were filtered out using our exclusion criteria. Seventy-eight full-text articles were selected for further investigation. After the intensive assessment, 23 papers were selected for evaluation. Finally, after group discussion and consensus, a total of 17 articles^3,8,13–27^ were chosen for data extraction and meta-analysis (Figure 1). A manual search for any additional relevant articles was not performed. There were 16 randomized trials and 1 retrospective study included. All the randomized trials were evaluated as low risk of bias. The retrospective study^27^ was accessed as ordinary quality by Newcastle–Ottawa Scale.^28^ Although the articles failed to present the details of randomization methodology and process, it is unlikely to induce severe influence to the results. The characteristics of studies are shown in Table 1.

**TABLE 1:**
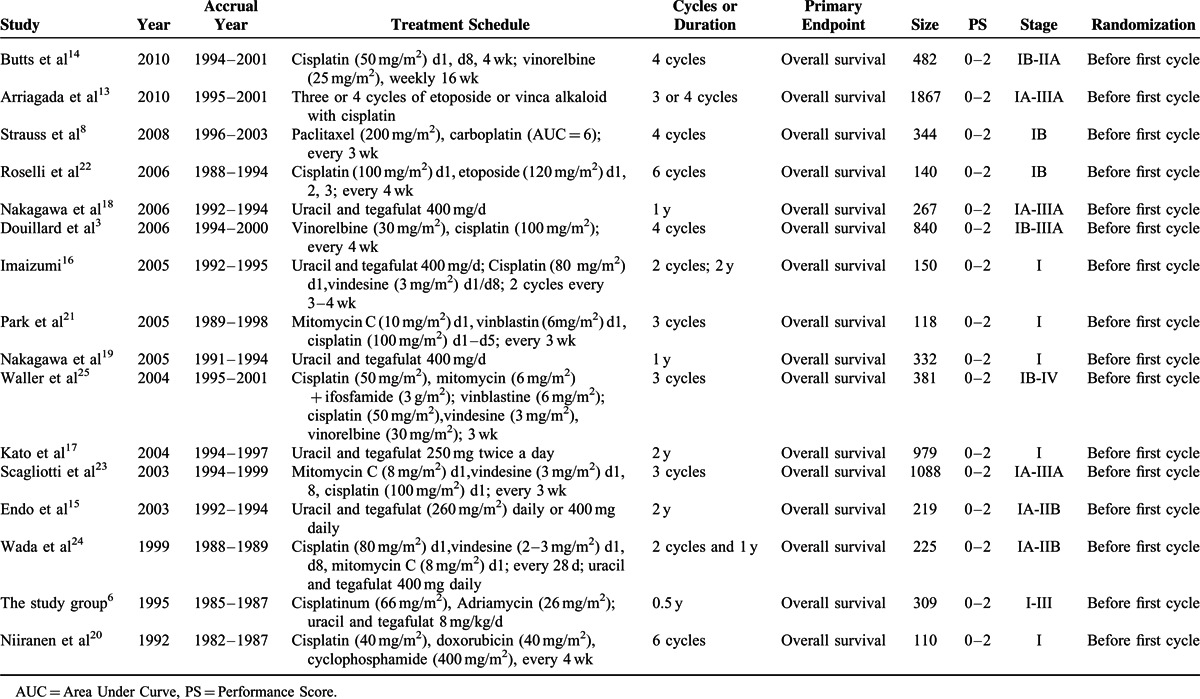
Characteristics of Included Studies for Meta-Analysis

All the 5-year OS and DFS data were extracted and recorded as “HR (95% CI)” and “N/M” (N = observed numbers of events, M = numbers randomized to the group). However, 2 articles^20,22^ reported the 10-year overall survival as the primary endpoint and were also recorded. Some studies did not present the HR directly but the *P* value of log-rank test and total observed events number.^8,16–19,21,22,27,29^ One of the relevant papers presented 90% CI.^8^ As a result, we used the method reported by Parmar et al and Tierney et al^30,31^ to calculate and verify their HRs and 95% CI.

We noticed a significant publication bias from the result of overall survival analysis as the Egger test *P* value was 0.038. We performed a sensitivity analysis and found that no publication bias existed either in Begg test or Egger test after excluding the data of Park's^27^ study. We decided to exclude Park's data from the analysis. There were 16 articles in the final analysis (Figure 1).

A total of 4656 patients were eligible for the meta-analysis. Among them, 2338 patients were assigned to the adjuvant chemotherapy group and 2318 were assigned to the control group (surgery alone). Characteristics of patients are demonstrated in Tables 2 and 3. The median age of these patients was 61. Men accounted for the largest proportion of all patients (72.5%), and the majority of all patients had a performance status of 0 (60.9%). The longest median follow-up time was 9.3 years,^14^ while the shortest median follow-up time was 3.36 years.^25^ Histology analysis demonstrated that squamous cell lung cancer accounted for 39.8%, while adenocarcinoma 48.2% and other or nonspecific for 9.2% of all included patients. In regard to the chemotherapy, 9 of 16 studies chose platinum-based therapy for patients in their treatment groups. Among these studies, 119 patients in 2 studies received 6 cycles of platinum-based chemotherapy,^20,22^ while 1143 patients in 7 studies received 4 or fewer cycles of platinum-based chemotherapy.^3,8,13,14,21,23,25^ Uracil-tegafur alone or in combination with platinum-based chemotherapy was chosen as the treatment regimen in 7 of the 16 included studies. Among them, 1 study had both a uracil-tegafur treatment group and a combined therapy (uracil-tegafur + platinum-based chemotherapy) group.^16^ In 5 studies from Japan, 884 patients were assigned to chemotherapy treatment group and received uracil-tegafur alone.^15–19^ Furthermore, 242 patients in 3 studies had received a combination of platinum-based therapy and uracil-tegafur.^16,24,26^

**TABLE 2:**
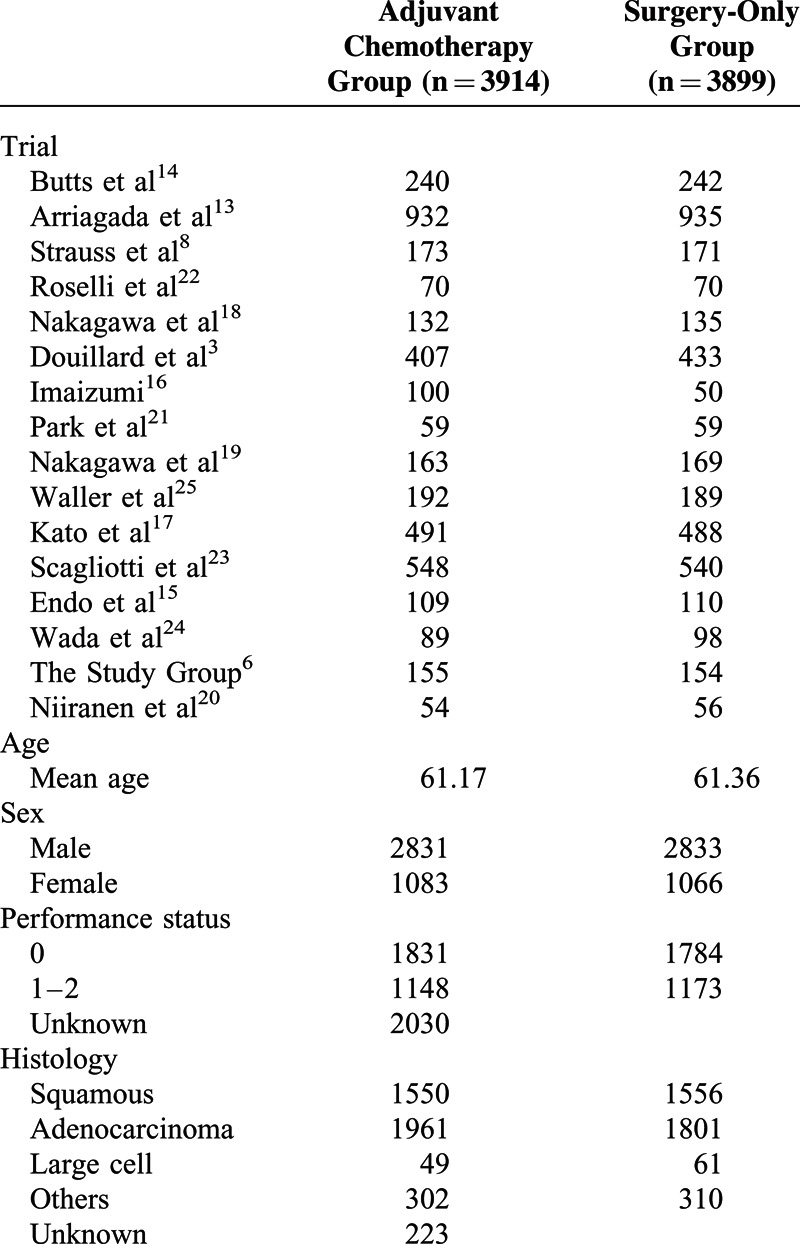
Characteristics of Included Studies for Meta-Analysis

**TABLE 3:**
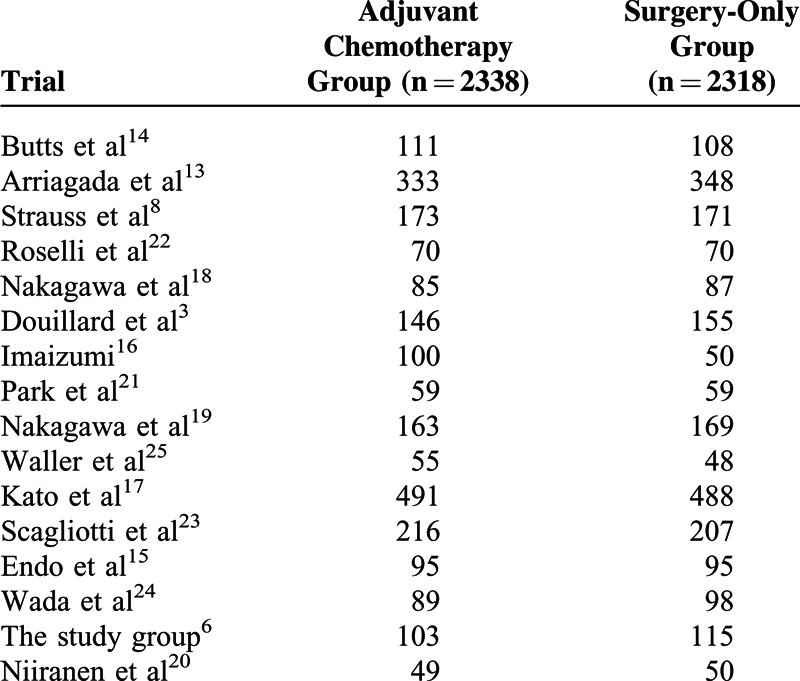
The Number of Stage IB NSCLC Patients in the Included Studies

### Overall Survival

**FIGURE 2:**
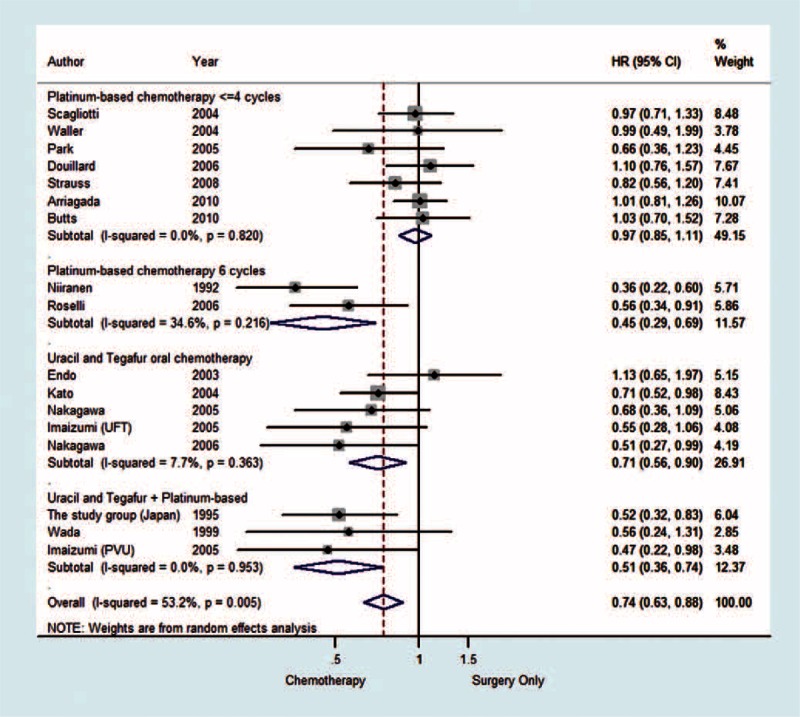
The subgroup analysis of OS in the completely resected stage IB nonsmall cell lung cancer patients who had received different chemotherapy and different therapy cycles. CI = confidence interval, HR = hazard ratio, OS = overall survival, PVU = cisplatin/vindesine+uracil/tegafur, UFT = uracil/tegafur.

The OS analysis demonstrated that the patients in the treatment group slightly benefited from adjuvant chemotherapy compared with patients in the control group patients (HR 0.74 [95% CI 0.63–0.88] *P* < 0.001). In the platinum-based chemotherapy group, an interesting result was observed. Patients who received 6 cycles of platinum-based therapy had better OS than those in the control group (HR 0.45 [95% CI 0.29–0.69] *P* < 0.001). However, patients who received 4 or fewer cycles of platinum-based therapy had no significant superiority in OS compared to the control group (HR 0.97 [95% CI 0.85–1.11] *P* = 0.664). In the uracil-tegafur oral chemotherapy subgroup, the treatment group had an obvious better overall survival rate than the control group (HR 0.71 [95% CI 0.56–0.90] *P* = 0.004). Patients who received the combination of platinum-based chemotherapy and uracil-tegafur also had a better OS than those in the control group (HR 0.51 [95% CI 0.36–0.74] *P* < 0.001) (Figure 2).

### Heterogeneity of Overall Survival

As shown in Figure 2, the heterogeneity of each subgroup was not significant (the *P* value is 0.82, 0.216, 0.363, and 0.953, respectively). There was a slight heterogeneity overall (I^2^ = 53.2%, *P* = 0.005), even though the random-effect model was selected. By performing the sensitivity analysis, we discovered that the results were consistent with the previous results after discarding the data that contributed the most heterogeneity.

### Disease-Free Survival

**FIGURE 3:**
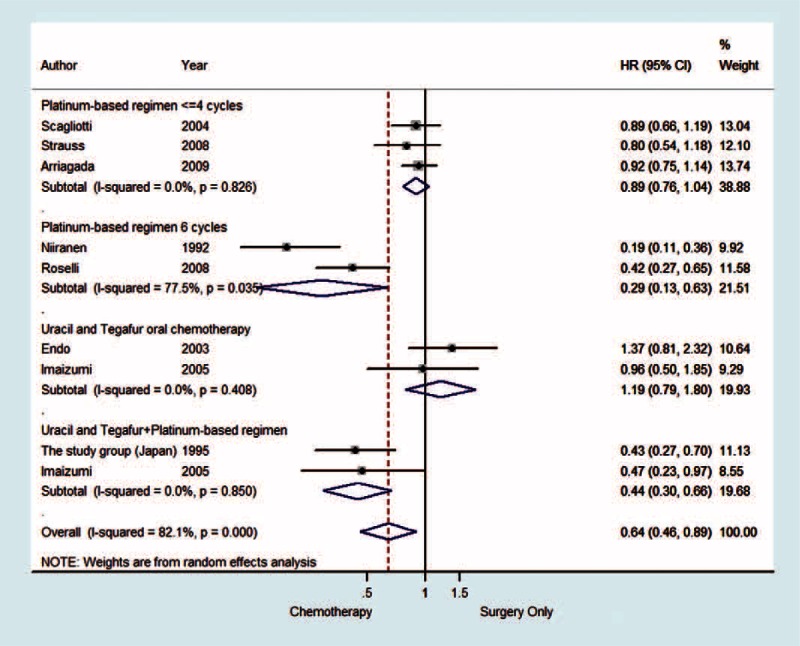
The forest plot of HR of DFS in the completely resected stage IB nonsmall cell lung cancer patients who had received different chemotherapy and different therapy cycles. CI = confidence interval, DFS = disease-free survival, HR = hazard ratio.

Only 10 of 17 articles were available and extracted for analysis. The random-effect analysis model was also utilized because of the heterogeneity. In the subgroup analysis, patients who received 4 or fewer cycles of platinum-based therapy had no advantage when compared to the control group (HR 0.89 [95% CI 0.76–1.04] *P* = 0.149). However, the DFS of the 6-cycle platinum-based therapy subgroup was significantly better than the control group (HR 0.29 [95% CI 0.13–0.63] *P* = 0.002). The platinum-based therapy in combination with uracil-tegafur treatment was superior to the control group (HR 0.44 [95% CI 0.30–0.66] *P* < 0.001). But patients who received only uracil-tegafur had no advantage compared to the patients in the control group (HR 1.19 [95% CI 0.79–1.80] *P* = 0.399) (Figure 3).

### Heterogeneity of Disease-Free Survival

Even though heterogeneity was not existent in any of the subgroups (I^2^ = 0.0%), except for the 6-cycle platinum-based group (I^2^ = 77.5%, *P* = 0.035). The overall heterogeneity was rather significant (I^2^ = 82.1%, *P* < 0.001). Nevertheless, our results showed that adjuvant chemotherapy had a slight advantage (HR 0.64 [95% CI 0.46–0.89] *P* = 0.008).

### Publication Bias

As we had mentioned above, the Begg and Egger test results indicated no publication bias in 16 relevant studies without Park's data. The results are shown in Table S1, http://links.lww.com/MD/A284.

## DISCUSSION

A total of 16 randomized trials were analyzed to determine via OS and DFS whether or not adjuvant chemotherapy is beneficial for stage IB completely resected NSCLC patients. Our analysis found adjuvant chemotherapy to be beneficial in patients with completely resected stage IB NSCLC in terms of OS and DFS. However, due to the overall heterogeneity of OS and DFS (I^2^ = 53.2% and 82.1%, respectively), it may not be a definite conclusion. Even so, we discovered some valuable information through the subgroup analysis process.

A number of clinical trials, meta-analysis, and NCCN guidelines recommended 4 to 6 cycles of platinum-based therapy for responsive patients.^32–35^ Rossi et al had performed a meta-analysis comparing 6 versus fewer planned cycles of first-line platinum-based chemotherapy for stage III-IV NSCLC patients^36^; the result demonstrated that there was no difference between 6 or fewer cycles in terms of OS and PFS. On the contrary, our study in the stage IB NSCLC patients found that patients who received 6 cycles of platinum-based therapy after surgery had better OS and DFS than those who only received only surgery. However, there was no clear evidence to indicate that patients who received 4 or fewer cycles of platinum-based therapy after surgery gain more benefits in OS or DFS than those who only received surgery. Park et al performed a retrospective study which reported that 4 cycles of platinum-based adjuvant chemotherapy brought benefit to the patients.^27^ However, the author indicated that the adjuvant chemotherapy was performed if the patients with risk factors such as poor differentiation, lymphovascular invasion, and pleural invasion. It was likely that patients with high risk factors would have worse prognosis. As a result, the efficacy of the adjuvant chemotherapy might have been overstated by the patient selection bias. Furthermore, it induced publication bias to the overall survival result, which was confirmed by Egger test. As a result, we decided to exclude this article from the analysis. However, it seemed that the result of the analysis would not be affected after this exclusion.

Moreover, we did an analysis of the same endpoints on postoperative stage I-III NSCLC patients. The results demonstrated that patients in both 6-cycle and fewer cycle subgroups gained benefits from the adjuvant chemotherapy. Data were shown in supplement (Figure S1, http://links.lww.com/MD/A284). It indicated that 4 or fewer cycles of adjuvant chemotherapy could bring benefit to the stage II-III NSCLC patients.

The HRs reported in the 6-cycle subgroup were for 10-year OS and DFS; HRs in other included articles presented only 5-year OS and DFS. It is possible that both subgroups have no advantage when comparing the 5-year OS or DFS to surgery alone, but have more long-term benefits such as prolonging 10-year OS and DFS. Although Mineo et al^29^ had reported that 6-cycle platinum-based therapy was superior to surgery alone in terms of 5-year OS and DFS (HR 0.5 and 0.475, respectively), the head-to-head comparison between 6-cycle and fewer cycle adjuvant chemotherapy was lacking. More clinical trials have to be performed to compare the effect of different cycles of platinum-based adjuvant chemotherapy in long-term survival.

Oral treatment of uracil-tegafur was firstly introduced to the public in 1979 by Japanese researchers.^37^ After that, more studies were conducted and it was discovered that oral administration of uracil-tegafur had an anticancer effect in several solid cancers. The West Japan Study Group for Lung Cancer Surgery reported that OS was longer in the treatment group (uracil-tegafur) patients than in patients assigned to the control (surgery alone) group after complete resection of stage I-III lung cancer.^24^ Our study results were the same as these previous studies for patients with resected stage IB NSCLC who received uracil-tegafur, except that there was no benefit in terms of DFS.

In 3 trials of uracil-tegafur plus platinum-based therapy in patients with stage IB lung cancer, the patients assigned to the treatment group had better OS and DFS than those assigned to the control group. The study reported by Imaizumi et al is a 3-arm trial containing uracil-tegafur, uracil-tegafur plus platinum-based therapy, and control groups.^16^ They reported that patients in both treatment arms had better outcomes than those in the control arm. In terms of the comparison between 2 treatment arms, it demonstrated that there was a slight advantage of uracil-tegafur plus platinum-based therapy, but not statistically significant. However, we were unable to confirm this finding through our own subgroup analysis because our own analysis only compared platinum-based chemotherapy combined with uracil-tegafur to our control group as there was not enough data present. Future studies are needed to explore the role of uracil-tegafur in combination with platinum-based therapy for patients with NSCLC.

A number of limitations have to be addressed in the present study. Firstly, some of the HRs were not directly reported in the texts so we had to calculate the HR from the observed patient numbers and *P* value of log-rank test. This method is not as accurate as the HRs reported by the authors. Secondly, the OS and DFS were calculated from the time after surgery in some studies, but from the date of diagnosis in other studies, which might prolong the OS. Thirdly, during the searching and evaluating process, we noticed that some studies included patients other than stage I; the data of stage I patients were unable to be separated from the data as a whole and were therefore discarded in accordance with our exclusion criteria. The loss of such data potentially weakens the reliability of our analysis results because of the loss of such data. Next, the OS and DFS Kaplan–Meier curves of all included patients were unable to be presented without the individual data. We analyzed the OS and DFS between different chemotherapy subgroups. If the individual data were available, more subgroup analysis could be performed, for example, gender, tumor size, age, toxicity, and smoking condition, which would add to precision of our study. Finally, all the studies of uracil-tegafur were performed on Asian people; other races were not included. Thus, a conclusive claim that uracil-tegafur is beneficial to all postoperative stage IB NSCLC patients is not able to be made.

In conclusion, platinum-based therapy is able to increase long-term OS and DFS of postoperative stage IB NSCLC patients. Patients who received postoperative 6-cycle of platinum-based chemotherapy, uracil-tegafur, or a combination of the 2 had better OS than the patients in the control group. However, patients who received only 4 or fewer cycles of platinum-based chemotherapy had no advantage comparing to the control group. For DFS, postoperative 6-cycle platinum-based chemotherapy alone or in combination with uracil-tegafur brought benefit to patients compared to the surgery group, but patients who were prescribed 4 or less cycles of platinum-based chemotherapy or uracil-tegafur alone had no advantage in prolonging DFS compared to the control group. Though a number of limitations exist in the present study, our findings provide potentially useful information; however, new randomized control trials are needed to make a more conclusive argument and to figure out the different effect of 6 cycles and fewer cycles of platinum-based chemotherapy and explore the effect, dosage, and toxicity of the regimens.
